# The Lectin Frontier Database (L*f*DB), and Data Generation Based on Frontal Affinity Chromatography

**DOI:** 10.3390/molecules20010951

**Published:** 2015-01-08

**Authors:** Jun Hirabayashi, Hiroaki Tateno, Toshihide Shikanai, Kiyoko F. Aoki-Kinoshita, Hisashi Narimatsu

**Affiliations:** 1 Research Center for Stem Cell Engineering, National Institute of Advanced Industrial Science and Technology, Central-2, 1-1-1, Umezono, Tsukuba, Ibaraki 305-8568, Japan; E-Mail: h-tateno@aist.go.jp; 2 Research Center for Medical Glycoscience, National Institute of Advanced Industrial Science and Technology, Central-2, 1-1-1, Umezono, Tsukuba, Ibaraki 305-8568, Japan; E-Mails: t.shikanai@aist.go.jp (T.S.); h.narimatsu@aist.go.jp (H.N.); 3 Department of Bioinformatics, Faculty of Engineering, Soka University, 1-236 Tangi-machi, Hachioji, Tokyo 192-8577, Japan; E-Mail: kkiyoko@soka.ac.jp

**Keywords:** L*f*DB, frontal affinity chromatography (FAC), lectin-oligosaccharide interaction, dissociation constant (*K*_d_), Pfam, scaffold, dynamic equilibrium

## Abstract

Lectins are a large group of carbohydrate-binding proteins, having been shown to comprise at least 48 protein scaffolds or protein family entries. They occur ubiquitously in living organisms—from humans to microorganisms, including viruses—and while their functions are yet to be fully elucidated, their main underlying actions are thought to mediate cell-cell and cell-glycoconjugate interactions, which play important roles in an extensive range of biological processes. The basic feature of each lectin’s function resides in its specific sugar-binding properties. In this regard, it is beneficial for researchers to have access to fundamental information about the detailed oligosaccharide specificities of diverse lectins. In this review, the authors describe a publicly available lectin database named “Lectin *f*rontier DataBase (L*f*DB)”, which undertakes the continuous publication and updating of comprehensive data for lectin-standard oligosaccharide interactions in terms of dissociation constants (*K*_d_’s). For *K*_d_ determination, an advanced system of frontal affinity chromatography (FAC) is used, with which quantitative datasets of interactions between immobilized lectins and >100 fluorescently labeled standard glycans have been generated. The FAC system is unique in its clear principle, simple procedure and high sensitivity, with an increasing number (>67) of associated publications that attest to its reliability. Thus, L*f*DB, is expected to play an essential role in lectin research, not only in basic but also in applied fields of glycoscience.

## 1. Lectins: A Diverse Group of Carbohydrate-Binding Proteins. Investigation and Controversies

Lectins, a key theme in this special issue, have a long history of investigation that stems back to the discovery in 1888 of the toxin ricin from the caster bean plant *Ricinus communis* [1]. Many plant lectins showing characteristic hemagglutinating activity have been identified, as evidenced in a recent review [2]. Therein, it was shown that lectins from *Phaseolus vulgaris* (bean), *Pisum sativum* (pea), *Lens culinaris* (lentil) and *Vicia sativa* (vetch) [2] have proved to be valuable tools for the analysis and separation of animal cells, and glycoproteins derived from them. As a protein family, L-type (L stands for legume) lectins form an extremely diverse group of compounds. However, many other lectin families have been identified in plant species, including R-type lectins, which are members of the ricin B chain-related lectins [3]. Interestingly animal lectins also have long history of investigation [4]. According to Kilpatrick, “Charcot-Leyden crystal protein” (CLCP), first identified in 1853 as crystal-like structures in pathological tissues [5], has now been recognized as a member of the galectin family (galectin-10) [6]. However, this designation does not seem appropriate, because CLCP binds mannose in “crystal” structures, but not β-galactoside in solution.

The first animal lectin “activity” was found in snakes in 1902 [7]. Before then, however, the rattlesnake *Crotalus durissus* was shown to contain both agglutination and lysis activity toward erythrocytes and leukocytes, and an article by Mitchell and Reichert in 1886 (two years before the discovery of ricin) contains a description of this fact [8]. The rattlesnake lectin is now known to belong to the C-type (calcium-dependent) lectin family [9], which, along with galectins, forms one of the largest protein families in the animal kingdom [10]. 

In this context, classic lectins, of which biochemical properties have been investigated mainly via probing their hemagglutination activity, are represented by a number of plant and animal lectins; *i.e.*, R-type [3], L-type [11], C-type [10], P-type (mannose-6-phosphate-binding) [12], and I-type (immunoglobulin-related) lectins [13], as well as galectins [14]. This kind of nomenclature (a single alphabet-type, with some exceptions, such as galectins) was first introduced by Drickamer, who attempted to classify C-type animal lectins in the particular context of comparison with another group of animal lectins known at that time as “developmentally regulated soluble β-galactoside-binding lectins” [15]. Based on the report that galectin-1 (the most investigated member at that time) requires a thiol (SH)-reducing reagent for the maintenance of activity, he proposed to name this group as “S-type,” referring to their supposed SH-requirement. However, this turned out later to be erroneous, as many other S-type lectins are not SH-requiring. As a result, a group of researchers in the field discussed the matter of renaming S-type lectins, and finally reached a consensus in 1994 that this group of lectins, which are evolutionarily conserved and have carbohydrate specificity for β-galactosides, should be designated “galectins” [16]. Thus, both the classification and designation have long been complicated issues in lectin research, both for plant and animal lectins [2,17].

## 2. Trends in Lectin Classification

### 2.1. Based on Specificity

For many years in the 20th century, lectins were classified according to their monosaccharide specificity; this was based on observations made with the hemagglutination inhibition test using simple saccharides (mostly monosaccharides and their derivatives). In 1994, Doyle *et al.* [18] listed 237 lectins from animal (61), plant (154) and microorganism (22) origins, which had been reported at that time. These were categorized into five groups based on monosaccharide specificity: *i.e.*, Gal/GalNAc (61%), Glc/Man (14%), GlcNAc (12%), L-Fuc (7%) and sialic acid (6%) [18]. The proportion of Gal/GalNAc-specific lectins appears to be high: this attests to the functional importance of Gal/GalNAc lectins [19], but one could also speculate that these lectins have advantages over other lectins in relation to the detection and purification tools that are available; e.g., lactose and asialofetuin-agarose, respectively. However, lectins with much more complex recognition profiles may be difficult to discover, or their properties may be difficult to rigorously define as would be required in a scientific paper.

Towards the end of 20th century, the status of lectin research was altered markedly with the increased availability of genome-related information. This took place first with regards to the nematode *Caenorhabditis elegans*, the first multicellular organism in which genome sequencing was accomplished [20,21,22]. For these genome-derived lectins (candidates), functional analysis was performed with recombinant proteins and advanced analytical methods, typically involving microarray techniques (for a recent review of the glycan array, see ref. [23]) that facilitated a much higher throughput than was possible with the conventional hemagglutination assay.

### 2.2. Based on Protein Family (Pfam)

Early attempts to classify lectins were made in a variety of ways, e.g., based on specificity, biochemical properties, biological distribution, *etc.* However, as described above, the course of lectin research changed greatly with the advent of genome hunting. Accordingly, the number of lectins discovered also increased significantly, and the properties of these lectins have now been elucidated in terms of functional genomics. Thus, lectins are now understood and classified from a more objective and systematic viewpoint. In this context, it seems reasonable to classify them on the basis of molecular structures (*i.e.*, protein families) combined with information available in genome databases. The protein family (Pfam) database contains information about protein domains and families, with Pfam-A forming the manually managed portion of this database that contains over 14,800 entries in the current release (version 27.0) [24]. It should be noted that not all members of a lectin-related Pfam are necessarily shown to have actual carbohydrate-binding properties: some can be non-lectin proteins, while others have not as yet been characterized as lectins. On the other hand, some classic lectin Pfams are composed almost entirely of lectin-proved members.

C-type lectins and galectins form the two largest lectin families in the animal kingdom, but it is also true that many homologues to classic plant lectins exist in animals. These include R-type [3], L-type [11], and jacalin-related lectins [25]. Descriptions of the properties [26] and three-dimensional structures [27] of all of these lectins have been reported in the literature. Recently, Fujimoto *et al.* reported on protein scaffolds of as many as 48 lectin families, for which three-dimensional structures and lectin functions have been reported in scientific papers [27]. This number however excludes carbohydrate-binding modules found uniquely on glycohydrolases, which often contain R-type lectin domains (Pfam: PF00652). Therefore, it seems that the number of lectin domains is likely to exceed 100.

## 3. Determination of Oligosaccharide Specificity of Lectins in Terms of Dissociation Constant (*K*_d_)

### 3.1. Methods to Determine K_d_

Various methods are available to quantitatively determine lectin specificity. They are represented by the following:

- Equilibrium dialysis
- Isothermal calorimetry (ITC)
- Surface plasmon resonance (SPR)
- Fluorescence polarization
- Frontal affinity chromatography (FAC)
- Capillary affinity electrophoresis

However, from a current glycomic viewpoint, it is important to consider that such a method should not only be accurate and reproducible, but should also have satisfactory throughput and speed. For these reasons, equilibrium dialysis is not appropriate for producing high-throughput data [28]. On the other hand, isothermal calorimetry (ITC) instruments are more advanced than before, and can provide thermodynamic parameters, such as Δ*H* and *S*, and consequently, Δ*G* [29]. However, the method requires substantial amounts of glycans for analysis and may therefore not be viable. Analysis based on a surface plasmon resonance principle has been widely attempted, but its application to small molecular glycans has a basic difficulty in terms of sensitivity [30]. Fluorescence polarization requires prior preparation of appropriately labeled glycan probes, to which non-labeled glycans are used as inhibitors [31]. However, a series of non-labeled glycans are not easily available. Capillary-based lectin affinity electrophoresis (capillary affinity electrophoresis) enables high-throughput and precise determination using a small amount of labeled oligosaccharides in a simultaneous manner [32]. However, the method requires technical expertise in capillary electrophoresis. 

Among the methods outlined above, frontal affinity chromatography (FAC) is unique in that it has a range of methods for detection; *i.e.*, radioisotope (RI) [33], mass spectrometry (MS) [34,35] and fluorescence detection (FD) [36,37,38,39,40,41]. To perform FAC-RI, however, *N*-glycans must be pre-radiolabeled, e.g., with NaB[^3^H]_4_. Similarly, for FAC-MS, modification of glycans with an appropriate alkyl reagent is necessary to increase the ionization efficiency in MS. On the other hand, FAC-FD is easily performed with a conventional high-performance liquid chromatography (HPLC) system toward a commercially available panel (>100) of pyridylaminated (PA)-glycans. Moreover, PA-glycans show excellent performance in their separation and sensitivity in FAC-FD (for review, see refs. [40,41]). To this end, the use of PA-glycans and FAC-FD affords sufficient sensitivity (<5 pmol/analysis) and reproducibility (CV < 5%). Of particular note is the fact that the PA-glycans show no detectable nonspecific adsorption on the resin, e.g., agarose. This is an important factor to determine *K*_d_ values precisely. Another labeling reagent, 2-aminobenzamide (2-AB), also shows adequate performance comparable to PA-glycans (unpublished observations) in terms of both sensitivity and non-specific adsorption, although standard 2-AB glycans are not readily available. 

**Figure 1:**
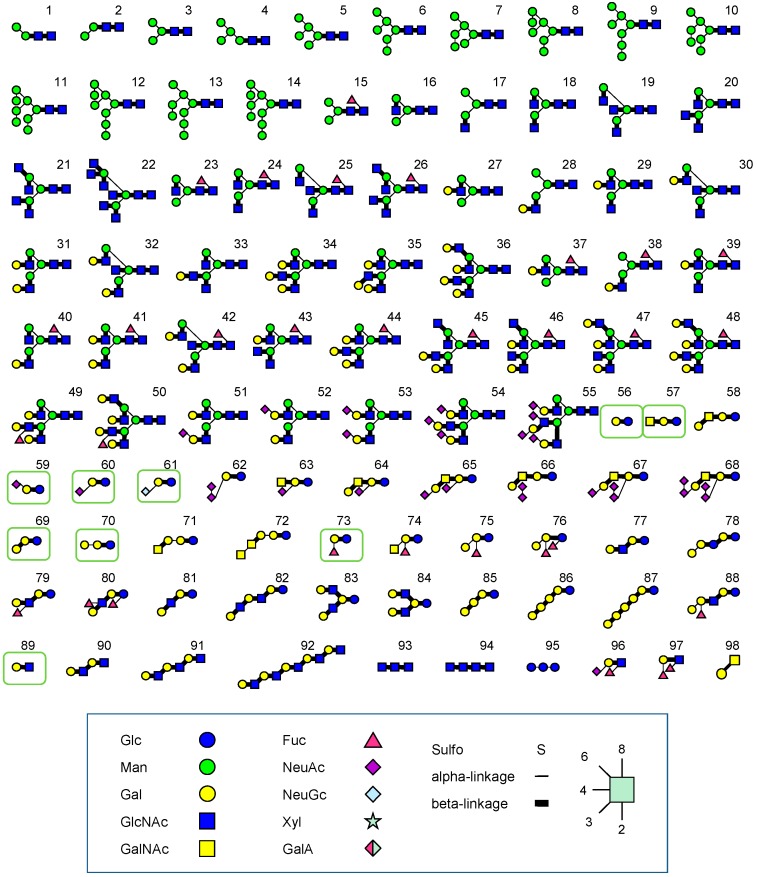
A list of pyridylaminated (PA) oligosaccharides routinely used for FAC. Nine oligosaccharides which are related to one another are described in the context of “One Parameter” function in the L*f*DB (see text and Figure 2). Note that reducing terminal end of PA-saccharide is opened as a result of monoamine coupling with 2-aminopyridine.

FAC-FD enables the systematic and reliable determination of *K*_d_ for immobilized lectins and a series of standard PA-oligosaccharides (see Figure 1 for PA-oligosaccharides used for routine FAC analysis). Since *K*_d_ values are considered to be inherent to individual lectin-glycan pairs at a given temperature (e.g., 25 °C), they are fundamental not only for the elucidation of lectin properties, but also for the interpretation of results obtained by lectin microarray, a novel platform for glycan profiling [42,43,44,45,46]. In practical use for glycan profiling, antibodies do not work for various reasons. Rather, lectins can do much better given their wider coverage as pointed out previously [47]. Therefore, datasets obtained by FAC are important for interpreting the results of lectin microarray analysis as well as those derived by other lectin technologies.

**Figure 2:**
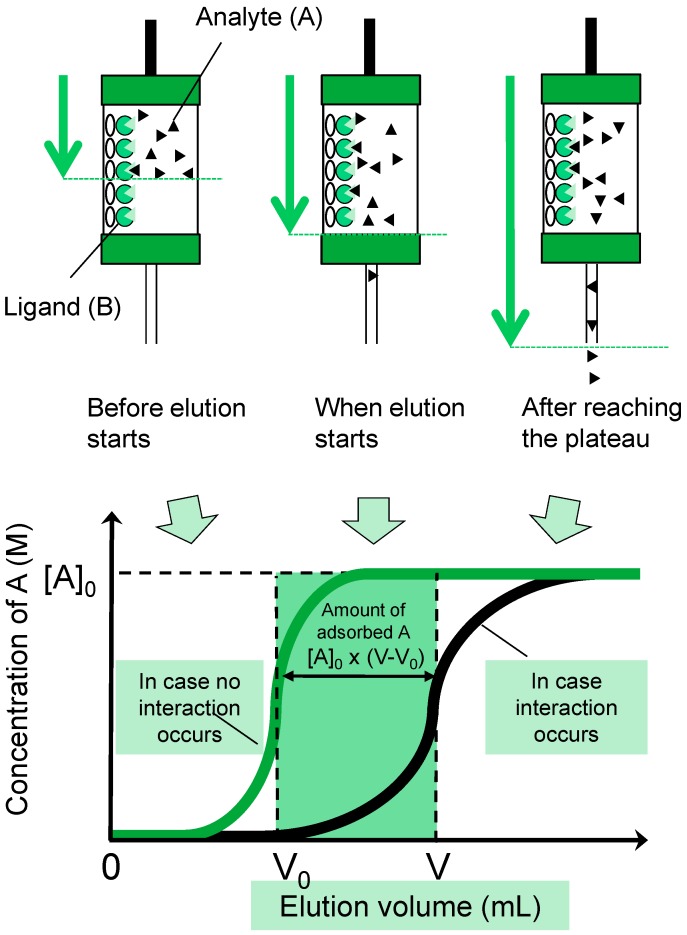
A schematic drawing of an FAC procedure and resultant elution curves.

### 3.2. FAC: Basic Principle and Procedures

**Figure 3:**
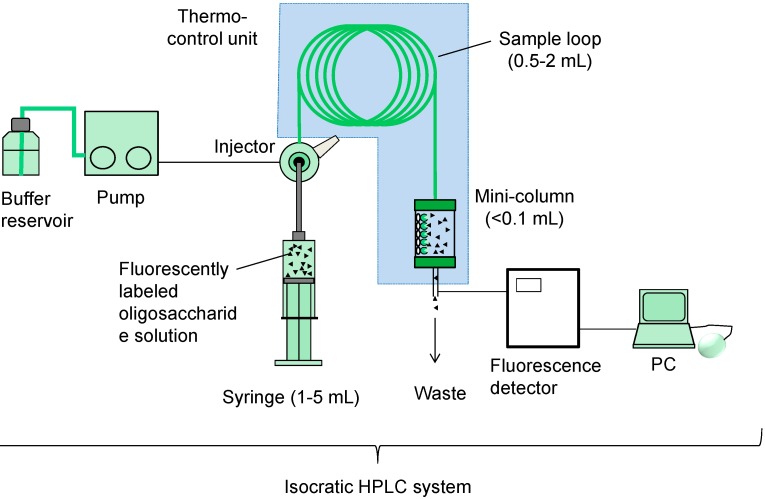
Fabrication of a basic FAC system. A conventional HPLC system can be used for FAC analysis, whereas a large sample loop (0.5–2 mL) relative to a column (0.1 mL or smaller) is used.

FAC was originally developed as a quantitative method by Kasai in 1976 [48]. The theory underlying FAC has been described in detail previously [49], and in recent reports by Kasai himself [50,51]. A standard scheme for the procedure is shown in Figure 2, for which the basic equation for FAC is expressed as follows:

*K*_d_ = *B*_t_/(*V* − *V*_0_) − [A]_0_ (1)

where *B*_t_ is the effective ligand content (expressed in mol) of a lectin-immobilized column, *V* and *V*_0_ are elution front volumes of analyte and a control substance, respectively, and [A]_0_ is the initial concentration of the analyte (e.g., PA-glycans). *B*_t_ should be obtained from another set of experiments under the umbrella of “concentration-dependence analysis” (see, Section 3.3). A recently developed automated system (FAC-1) is equipped with a pair of capsule-type miniature columns (each 2.0 mm in diameter and 10 mm in length, with a bed volume of 31.4 μL) in line with a fluorescence detector (Shimadzu RF10AXL: for details, see [40,41]). Manual operation is also possible if a standard isocratic HPLC system is available (see Figure 3 for basic production of a FAC system), while a large sample loop (e.g., 2 mL) relative to column size (e.g., 0.1 mL) should be used. For this purpose, the use of commercially available gourd columns (4.0 mm in diameter and 10 mm in length, bed volume 125.6 μL) is recommended [37]. Typical conditions in the automated FAC-1 system are as follows: analytical speed, 5 min/analysis; sample requirement, 0.3–1.0 mL of PA-glycan solution (5–10 nM); resolution (experimental error), 3–5 μL in *V*–*V*_0_. In most cases, *K*_d_ values for lectins and glycans (10^−3^ to 10^−7^ M) are much larger than [A]_0_ (5–10^−9^ M), meaning that Equation (1) can be simplified to:

*K*_d_ = *B*_t_/(*V* − *V*_0_) (2)

As Equation (2) does not include [A]_0_, *K*_d_ values no longer depend on experimental errors related to the PA-glycan concentration, which is why FAC can provide precise and reproducible analysis distinct from other methods.

To summarize the advantage of the FAC-FD method over others [36,40,41]: (1) the principle is clear (based on a Langmuir’s adsorption law); (2) Even weak interactions such as those between lectins and glycans can be determined; (3) Only a small quantity (<5 pmol) of fluorescently labeled glycans is required; (4) Combined with an HPLC system, high-throughput analysis is easily achievable (a series of *K*_d_’s can be obtained once B_t_ is determined by concentration-dependence analysis); (5) The analysis is highly reproducible due to simple isocratic elution as well as independence from [A]_0_ according to Equation (2). To our knowledge therefore, FAC-FD is the only method that enables both the quantitative and high-throughput determination of lectin-glycan interactions in terms of *K*_d_. Despite these advantages, possible drawbacks should also be mentioned: (1) immobilization of lectins on agarose or other matrices may result in modification or reduction of their original binding ability, as is expected for some sialic acid-binding lectins, of which lysine residue(s) in their presumed sugar-binding sites may be damaged by a standard NHS (*N*-hydroxysuccinimide)-coupling procedure; (2) Even using a miniature column (2 × 10 mm, 31.4 μL), a relatively large amount of lectins (e.g., approximately 500 μg) is required to accomplish the total analysis; (3) A crude sample cannot be directly applied to the system, in contrast to FAC-MS which performs better for the analysis of mixed samples with different molecular masses [34] (*note* that analysis of a mixture of a fluorescently labeled target glycan with various concentrations of the non-labeled glycan is possible in FAC-FD); (4) for the determination of B_t_, a substantial amount of saccharide derivatives is necessary (usually *p*-nitrophenyl, *p*-methoxyphenyl or methotrexate derivatives are used for this purpose) [40,41].

As a result of comprehensive interaction analysis with >100 lectins and >100 glycans, which started in 2003 as part of a national project in Japan [52], the authors have systematically determined the interactions of these lectins and glycans in terms of *K*_d_. It was not easy to determine *K*_d_ values for some lectins because of a lack of appropriate sugar derivatives (e.g., *p*-nitrophenyl) required for the concentration-dependence analysis (described below). Even for such cases, however, relative affinity can be calculated according to Equation (2) (*note* that *K*_a_ = 1/*K*_d_, and thus, is proportional to *V* − *V*_0_). An apparent consequence of the FAC-FD method is the re-investigation of lectin specificity (*i.e.*, “visiting old, learning new”). This is evident from a GlcNAc-binding plant lectin from *Griffornia* (now reclassified *Bandeiraea*) *simplicifonia*, GSL-II, whose detailed sugar-binding specificity had not been elucidated before our FAC analysis was undertaken: FAC-FD analysis revealed that GSL-II recognizes, in a highly specific manner, a GlcNAc residue transferred by the action of GlcNAc transferase IV [53]. Other examples of re-investigation of lectin specificity include galectins. Through these studies, a consensus rule for galectin recognition was found: an empirical “Galβ-equatorial” rule for galectin-recognition disaccharides was derived [36]. However, this rule was not valid for a newly discovered nematode disaccharide, “Galβ1-4Fuc” [54], because this glycosidic linkage is directed to “axial” 4-OH of L-Fuc. After careful reconsideration of the structural data, the authors reached a decisive rule, *i.e.*, under the re-defined configuration “Galβ-(syn)-gauche” [38]. The rule proved to work perfectly for the differentiation of galectins from other types of lectins. Thus, new information can be gained by the re-examination of old results using FAC, which maintains and strengthens its role as a powerful tool investigating the sugar-binding specificity of novel lectins. Lectins so far analyzed by FAC-FD are summarized in Table 1.

**Table 1:** Summary of lectins, for which comprehensive quantitative analyses by FAC-FD have been made.

Lectin Categories	Pfam	Protein Fold	Lectins and Special Glycans Analyzed	Ref.
L-type (L-type-like)	PF00139	β-sandwich (jellyroll)	*Whisteria* (WJA, WFA, WBA)	[55]
			*Pisam sativum* (PSL)	[56]
			ERGIC-53, VIPL, VIP36	[57]
			*Erythrina crystagalli* (ECL) and other *Erythrina* species	[58]
			*Griffonia simplifolia* (GSL-II)	[53]
			*Wisteria floribunda* (WFA)	[59]
			VIP36	[60]
Galectin	PF00337	β-sandwich (jellyroll)	Mammalian galectins	[61]
			Fungal galectin (ACG)	[62]
			Mutants of nematode galectin LEC-6	[63]
			Conger eel (congerin P)	[64]
			Marine sponge *Halichondria okadai*	[65]
			Nematode gene DC2.3	[66]
			Mutants of ACG	[67]
			Two lectin domains of nematode galectin LEC-1	[68]
			Human galectins 1-9 and other lectins	[38]
			Nematode galectins	[69,70]
			American bullfrog *Rna catesbeiana*	[71]
			Cysteine-less mutant of human galectin-1	[72]
			Nematode galectin (LEC-6)	[73]
			Nematode galectins (LEC-1~LEC-11)	[74]
			Human galectins	[75]
			Marine sponge *Halichondria okadai*	[76]
			Mutants of nematode galectin (LEC-1)	[77]
			Human galectin-9 N-terminal CRD	[78]
			The nematode galectin LEC-1 and its mutants	[79]
			Argasid tick *Ornithodoros moubata*.	[80]
			*Xenopus* galectin-VIIa	[81]
			Human, chicken, nematode, sponge, fungal galectrins	[36]
			Human galectin-9	[82]
			Nematode galectin LEC-1 and N- and C-CRDs	[83]
C-type	PF00059	C-type α/β-fold	Atlantic salmon serum	[84]
calcium-dependent			Sea cucumber lectin CEL-IV	[85]
			DC-SIGN, DC-SIGNR, and LSECtin	[86]
			Langerin to sulfated and mannsylated glycans	[87]
			MGL1, MGL2, and their mutants	[88]
			Atlantic salmon C-type lectin receptor C (SCLRC)	[89]
			Acorn barnacle *Megabalanus rosa*, *Balanus rostatus*	[90]
R-type	PF00652	β-trefoil	Mutants of earthworm lectin EW29Ch	[91]
ricin B chain-related			RCA120/RCA-I	[58]
			Phytopathogenic *Sclerotinia sclerotiorum*	[92]
Jacalin-related	PF01419	β-prism I	Human ZG16 p	[93]
			Mannose-binding type Jacalin-related lectins	[37]
			Jacalin	[94]
			*Castanea crenata* agglutinin (CCA)	[95]
			*Cycas revoluta* leaf lectin (CRLL)	[95]
GNA-related monocot-type	PF04152	β-prism II	Two-domain GNA-related lectins	[96]
Sea urchin egg	PF02140	α/β-fold with two long	Shishamo smelt *Osmerus (Spirinchus) lanceolatus*	[97]
lectin (SUEL)-related or Rha-binding		structural loop	Ayu *Plecoglossus altivelis*	[98]
			Pearl shell *Pteria penguin*	[99]
P-type	PF02157	P-type α/β-fold	Mammalian ER lectin XTP3-B	[100]
mannose 6-phosphate receptor homology (MRH)			Gene *OS-9* (osteosarcoma-9)	[101,102]
Malectin	PF11721	β-sandwich (jellyroll)	ER-resident lectin	[103]
Fungal fucose-specific	PF07938	6-bladed β-propeller	Mushroom *Pholiota squarrosa*	[104]
			*Aspergillus oryzae*	[105]
AAL-like				
Fungal fruit body lectin	PF07367	α/β-sandwich	*Agaricus bisporus* agglutinin (ABA)	[106]
Others			Mussel, *Mytilus galloprovincialis*	[107]
orphan or family			Mushroom *Hygrophorus russula*	[108]
unidentified			Eggs of Japanese sea hare *Aplysia kurodai*	[109]
			Feather star *Oxycomanthus* *japonicas*	[110]
			Coronate moon turban *Turbo* (*Lunella*) *coreensis*	[111]
			Mushroom *Boletus venenatus*	[112]
			Red alga *Hypnea japonica*	[113]
			Pacific annelid *Perinereis nuntia* ver. *vallata*	[114]
			Mushroom *Boletopsis leucomelas* (BLL)	[53,115]

### 3.3. Determination of B_t_ (Effective Ligand Content) in FAC

In parallel to FAC interaction analysis using a series of fluorescently labeled (e.g., PA) glycans (Figure 1) as described above, a concentration-dependence analysis should be carried out for the determination of an effective ligand content (*B*_t_) for an individual lectin-immobilized column. For this purpose, either *p*-nitrophenyl or *p*-methoxyphenyl derivatives of simple saccharides are usually used (e.g., lactose-β*-p*NP), where their elution is detected by UV (280 nm). The concentrations used depend on the affinity between the immobilized lectin and the labeled saccharide: in theory, when [A]_0_ is the same as *K*_d_ expressed in molar, M, a *V* − *V*_0_ value is obtained that corresponds to one half of the maximal retardation of (*V* − *V*_0_), *i.e.*, *V*_Max_ − *V*_0_, where *V*_Max_ is defined as a *V* value when minimal [A]_0_ is used (*i.e.*, [A]_0_ << *K*_d_):

*V* − *V*_0_ = *B*_t_/(*K*_d_ + [A]_0_) (3)

If [A]_0_ << *K*_d_:

*V*_Max_ − *V*_0_ = *B*_t_/*K*_d_ (4)

Therefore, when [A]_0_ *= K*_d_, Equation (3) should simplify to:

*V* − *V*_0_ = *B*_t_/2*K*_d_ = (*V*_Max_ − *V*_0_)/2 (5)

**Figure 4:**
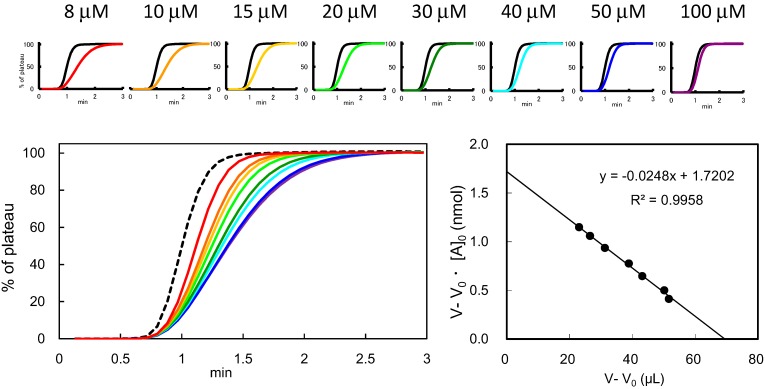
A typical case of concentration-dependency analysis using an ECA column. A series of diluted solution (8–100 μM) of *p*-nitoropheyl derivative of *N*-acetyllactosamine (LacNAcβ-pNP) are prepared, and are continuously applied to the column (2 × 10 mm) at a flow rate of 0.125 mL/min. The obtained *V* − *V*_0_ data are used for the determination of *B*_t_ (effective ligand content, nmol) of the column and *K*_d_ for LacNAcβ-pNP by the procedure of Woolf-Hofstee-type plots. *B*_t_ is obtained graphically as the intercept of y axis (in this case, 1.72 nmol), whereas *K*_d_ is obtained as the slope of the line (24.8 μM).

On this basis, concentration dependency analysis should ideally be performed with an [A]_0_ around the *K*_d_ value, with these concentrations being empirically in the range of 1 μM to 100 μM. Hence, a series of diluted saccharide solutions are applied to a lectin-immobilized column. Woolf-Hofstee-type plots are made for the *V* − *V*_0_ values obtained using these saccharide solutions. In Figure 4, a typical case is shown for an *Erythrina cristagalli* agglutinin (ECA), where an ECA-agarose column (3.0 mg/mL gel) is used, to which a series of diluted solutions of lactose-β*-p*NP (8–100 μM) is applied.

## 4. Lectin *Frontier* Database (L*f*DB)

### 4.1. Overview of the Database Contents

Lectin Frontier DataBase (L*f*DB: http://jcggdb.jp/rcmg/glycodb/LectinSearch?doc_no=1) has been constructed within the framework of the Japan Consortium for Glycoscience and Glycotechnology (JCGG: http://www.jcgg.jp/index_e.html) under the concept that those scientists who have been separately funded by the different ministries in Japan have joined this consortium and provide support through their own research grants. L*f*DB consists of (1) the Lectin Information Page to provide basic information about lectins and (2) the Interaction Page to provide the interaction data obtained by the FAC-FD system in terms of affinity constants (*K*_a_ = 1/*K*_d_). Interaction data are shown in a bar graph format by either an actual measurement (*V* − *V*_0_) or *K*_a_. L*f*DB also provides a “One-parameter” function that helps users to find a key structural element among the related glycan structures. To the present, 181 lectins have been registered in the database and FAC-FD data are available for 47 lectins. Table 2 summarizes the current content of L*f*DB.

**Table 2:** Summary of lectin entries in the current Lectin *f*rontier DataBase (L*f*DB).

Family (Entries)	Pfam ID	Origin	Monosaccharide Specificity (Entries)	FAC Data Deposited
Annexin (12)	PF00191	Mammals, protozoa, *etc.*	Others (12)	None
Chitin-binding (9)	PF00187	Plants, mammals	GlcNAc (8)	PL/STL, PWM, LEA/LEL, UDA, DSA/DSL, WGA
C-type (38)	PF00059	Mammals, invertebrates	Fuc (5)	None
			Gal/GalNAc (7)	None
			GlcNAc (3)	None
			Man/Glc (7)	None
		bacteria, virus	Others (2)	None
Fucose-binding (3) *	PF00754	Fish	Fuc (1)	None
	PF07938	Fungi	Fuc (1)	AAL
	PF07472	Bacteria	Fuc (1)	None
Galectin (26)	PF00337	Mammals, amphibian, fish,	Gal (23)	Xgalectin VIIa, C14, C16,
		Invertebrates, fungi	Gal (3)	GC1, GC2, ACG
Jacalin-related (19)	PF01419	Plants	Gal (3)	Jacalin
			Man/Glc (13)	BanLec, Conarva, CRLL, CCA, MornigaM, Calsepa, Heltuba, KM+/Artocarpin
Legume lectins (37)	PF00139	Plants	Fuc (2)	LTL, UEA-I
			Gal/GalNAc (16)	ECA/ECL, EcorL, ECafL, ELysL, EFlaL, DBA/DBL, BPA, BPL, PTL-I, PNA, SBA/SBL, VVL, WFA
			GlcNAc (1)	BSL-II/GSL-II/GS-II
			Man/Glc (9)	PSA/PSL,LCA/LCL/LcH
			Others (2)	PHA-E, PHA-L
Man-6Pi-binding (3)	PF02157	Mammals	Man-6Pi (3)	None
Monocot (15)	PF01453	Plants	Man/GlcNAc (11)	HHL, GNA/GNL, NPA/NPL, TxLCI
			unknown (4)	None
R-type (19)	PF00652	Mammals, invertebrates	Gal/GalNAc (11)	RCA-I/RCA120, EW29-Ch
		Plants, bacteria	NeyAc (4)	SSA, SNA
			Others (4)	None

Notes: * These three fucose-binding lectins registered in the current L*f*DB have now been classified into different protein families as shown in the column of Pfam ID; e.g., PF0075, PF07938, PF07472.

### 4.2. How to Use LfDB

The basic interface of the database is as follows:

Type keyword in the “Search” function page on the *left*, or choose categories (either Lectin family, Monosaccharide Specificity, or 3D-fold) in the “Classification” page. Click “Show All” to view the full lectin list.

**Figure 5:**
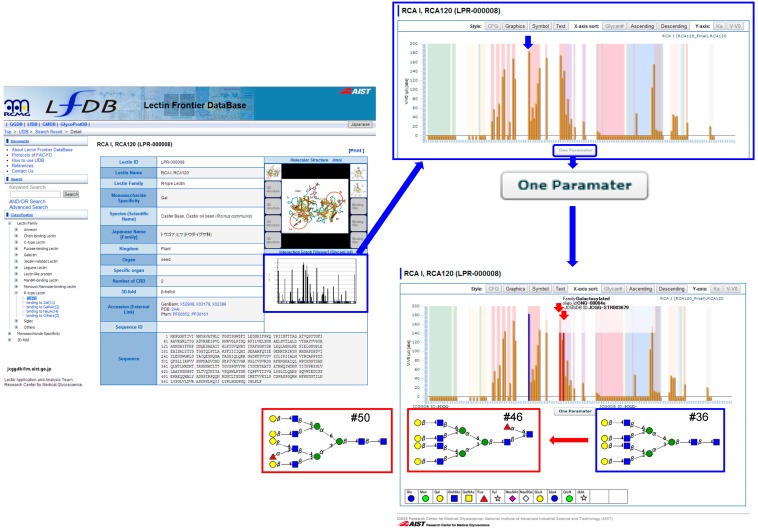
A flow of specificity analysis using “One Parameter” function. *Left* panel: As an example, RCA I/RCA120 is chosen, which belongs to the R-type lectin. Click the middle-right figure (boxed with *blue* line) to show the bar graph FAC data. *Right* panel (*top*): Select a particular oligosaccharide of interest: in this case the one showing the highest affinity (shown with *blue* short arrow). Click “One Parameter” button to show related but differ regarding one parameter: in this case, two oligosaccharides, #46 and #50 are shown (with *red* short arrow), which are α1-6-fucosylated in the reducing terminus and α 1-2-fucosylated in the non-reducing terminus, respectively, relative to the original oligosaccharide #36.

Click the lectin name to go to the “Lectin Information” page, or the Interaction Data to go to the “Interaction” page. When necessary, click “GlycanList” in the *right* top to download the glycan list used in the analysis. From the Lectin Information page, move to the Interaction page by clicking the “Viewer” button (Figure 5, *left*).

### 4.3. One-Parameter Difference Analysis

**Figure 6:**
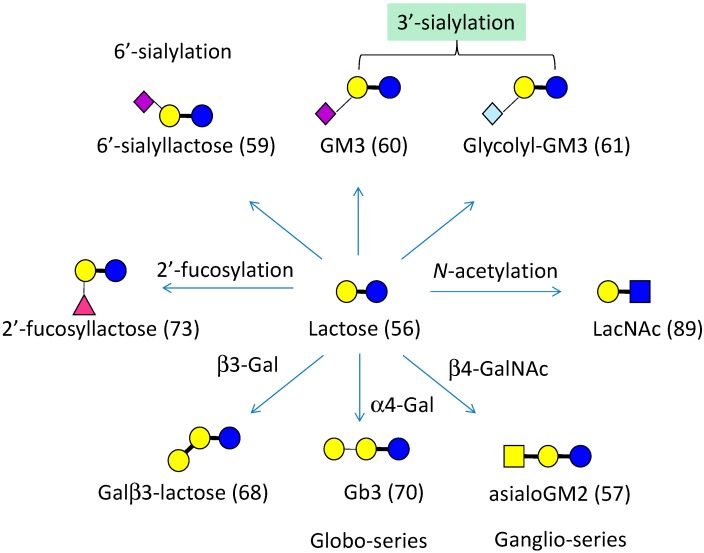
Lactose and its derivatives with “One Parameter” difference.

One-parameter difference analysis is a search tool for related glycan structures of a particular glycan. This is a very helpful function for determining a key feature of lectin recognition. For example, lactose (Galβ1-4Glc; #56) can be converted to related structures by the one-parameter difference option (Figure 6): *N*-acetylation to LacNAc (#89), α2-fucosylation to 2'-fucosyllactose (#73), β3-galactosylation to Galβ3-Lac (#68), α4-galactosylation to Gb3 (#70), β4-*N*-acetylgalactosamination to asialoGM2, 3'-sialylation to GM3 (#60, #61), and 6'-sialylation to 6'-sialyllactose (#59). These form a related group with respect to lactose. By comparison of before and after modification (e.g., *N*-acetylation, 2'-fucosylation) outcomes, it becomes clear which OH group is critical for recognition, and which substitution is effective to enhance affinity. In Figure 5 (*right*), an example of RCA-I is shown as a bar graph, which demonstrates the best affinity to a tetraantennary complex-type *N*-glycan (#36, *blue*-boxed). If this glycan is chosen, and the one-parameter button is clicked, two related glycans are indicated (*red* box below): one is a core α1-6 fucosylated glycan (#46) and the other is a Lewis X-type fucosylated glycan at the GlcNAc transferase IV-transferred GlcNAc residue (#50). Glycan #46 shows comparable affinity to RCA120, while glycan #50 shows a substantial decrease in affinity following Lewis X-type fucosylation. Therefore, it is possible to speculate that RCA120 requires 3-OH group of GlcNAc in the LacNAc recognition unit. This in fact proved to be the case in our detailed specificity analysis of RCA120 [58].

## 5. Future Plan for Improving the L*f*DB

At present, L*f*DB contains 181 lectin entries, among which 41 FAC datasets are available and accessible in terms of one-parameter function analysis. However, lectin research has progressed rapidly in recent times, and the analysis data also include those obtained by FAC analysis performed in other laboratories. In this regard, L*f*DB will be updated in a more comprehensive and sophisticated fashion: not only by increasing the numbers of lectins and FAC datasets but also by implementing the data using Semantic Web technologies [116]. In particular, the lectin and binding affinity data will be updated to use the Research Description Framework (RDF), so that all the data can be linked with related information in other databases. By doing so, it will become possible to expand the data with glyco-gene and related protein information. For example, since the tertiary structure data of PDBj has been converted to RDF, the annotations in PDB can potentially be easily found from L*f*DB. Moreover, details about glycan structures that bind strongly to a particular lectin can also be linked easily with existing glycan databases. Thus, by using the latest informatics technologies, an increased understanding of lectins and glycans can be gained, thus helping to elucidate their functions in complex cellular environments [116].
